# Revealing complex mosquito behaviour: a review of current automated video tracking systems suitable for tracking mosquitoes in the field

**DOI:** 10.1186/s13071-025-06666-6

**Published:** 2025-02-21

**Authors:** Beatrice H. Bredt, Frédéric Tripet, Pie Müller

**Affiliations:** 1 Swiss Tropical and Public Health Institute (Swiss TPH), Allschwil, Switzerland; 2 University of Basel, Basel, Switzerland

**Keywords:** Vector control, Mosquito, Behaviour, Experimental hut, 3D video tracking

## Abstract

**Abstract:**

Mosquito-borne pathogens continue to cause tremendous suffering, morbidity and mortality. For many of these diseases, vector control remains the most effective approach. The development and deployment of effective and efficient mosquito control products and strategies require a profound understanding of mosquito behaviour. To study complex mosquito behaviour, automated video tracking of mosquito flight paths has proven to be a comprehensive approach, and several video tracking approaches have emerged in recent years, making the choice for a suitable system challenging. Here, we conducted a literature review by searching PubMed and Google Scholar, and we identified 66 publications focusing on mosquito video tracking, which made use of eight different systems. We then compared and scored those video tracking systems by assessing their performance in the laboratory as well as their potential suitability for tracking mosquito behaviour in a field setting. While all eight systems have produced valuable information on mosquito behaviour, for tracking mosquitoes in the field, ‘Braid’, ‘EthoVision XT’ and ‘Trackit3D’ appear to be the most suitable systems as they need small disk capacity and are well adaptable to different settings. However, the optimal choice will ultimately depend on the specifications required to answer a given research question, the financial resources available and user preferences.

**Graphical Abstract:**

**Figure:**
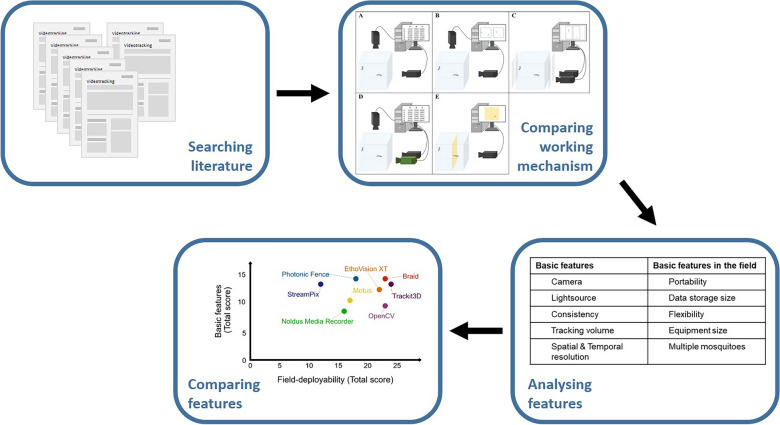

**Supplementary Information:**

The online version contains supplementary material available at 10.1186/s13071-025-06666-6.

## Mosquito control and video tracking

Mosquitoes are important vectors for a wide range of pathogens that infect both humans and animals, causing a heavy burden on human society [1]. The key mosquito-borne pathogens are the *Plasmodium falciparum* malaria parasite and dengue virus. For malaria, three main interventions have contributed to the drastic decrease in malaria incidence across the African continent over the past two decades [2]. The interventions include improved treatments of the human stages of the parasites through artemisinin-based combination therapy (ACT), insecticide-treated bed nets (ITN) and indoor residual spraying (IRS). Unfortunately, efforts to further decrease malaria cases have stalled in recent years, presumably because of insecticide resistance and limited funds [3]. Until an effective vaccine is available against malaria, the best malaria prevention method at the community level remains vector control [4]. Similarly, dengue control relies heavily on vector control because of the absence of a specific treatment and a widely deployable vaccine.

Insecticide-treated nets and IRS are evaluated in experimental hut trials conducted according to the World Health Organization (WHO) guidelines [5] to test their efficacy. Efficacy is assessed based on the reduction in the number of dead and blood-fed mosquitoes, expressed as the odds ratios between the test formulation and its comparator. While these ratios provide an overall measurement of efficacy, they do not reveal the underlying behaviour that has led to the observed outcome. This information could, however, be crucial in improving the delivery and design of an intervention. For example, mosquitoes may preferentially land on certain areas of an ITN [6] or IRS-treated wall [7], allowing for targeted treatment. Therefore, insight into mosquito behaviour provides the basis to improve vector control.

A particularly comprehensive and powerful approach to study mosquito behaviour is automated video tracking of mosquito flight paths [8], which began in the early 2000s [9]. Thanks to increased affordability and advancements in computing power, new video systems have been developed to address a broad range of research questions [10].

The video systems may capture various behaviours, such as flight manoeuvres [11], wing beat patterns [12], swarming behaviour [13–16], and how mosquitoes interact with ITNs [17, 18]. Using video tracking, Parker et al. [17] found that malaria mosquitoes mostly interact with the roof of an ITN, and it has been predicted that deploying mosaic nets with a roof treated with a different insecticide than the side panels could effectively decrease malaria transmission [19]. Tracking mosquito flight paths has also revealed the mosquitoes’ entry and resting behaviour in an experimental hut [20, 21], which could inform how to optimally deploy IRS, spatial repellents, and insecticide-treated eave tubes [22]. Automated video tracking may also lead to improved designs of mosquito traps by understanding how mosquitoes respond to human odour and heat [11] as well as their flight dynamics around them [23–25]. Thanks to video tracking, it has been shown that γ-rays-sterilised male mosquitoes do not exhibit reduced fitness and even show a higher flight performance [26]. Video tracking has also revealed more generally how mosquitoes respond to olfactory cues [11, 27–34] and temperature [27, 35–37].

While automated video tracking provides insights into mosquito behaviour, numerous hardware and software implementations exist, making the right choice for the most suitable system difficult, particularly for applications outside the laboratory environment. To facilitate the selection of an adequate automated system for tracking mosquitoes in the field—or the semi-field hut for the purpose of this review—we examined the existing literature on commercially available products and other accessible systems and assessed the suitability of these video tracking systems.

### What are the requirements for an automated video tracking system to track mosquitoes?

Irrespective of the context, an automated video system needs to show certain basic features to track a free-flying mosquito in space and time. The mosquito’s position, preferably in 3D, defined by its *x*-, *y*- and *z*-coordinates as a function of time (*t*), needs to be reliably tracked across the space of interest (i.e. the flight arena) with a spatial and temporal resolution providing enough detail to answer a given research question. The maximum area or volume that a system is able to cover depends on the cameras' field of view, depth of field and resolution, while the temporal resolution mainly depends on the camera’s frame rate and the computer processor. Since many mosquito vector species show a nocturnal biting behaviour [38], the cameras also need to be able to operate in natural darkness (i.e. in infrared light [39]). Moreover, to increase reproducibility and comparability of results within and between studies, it would be desirable that a system and its handling remain consistent across settings.

Tracking mosquitoes requires more than simply recording them on video, they need to be detected and identified via a computer algorithm. The most common technique is background subtraction in which moving objects (i.e. the mosquitoes) are detected by frame differencing, if the background remains static [40, 41]. A challenge with this approach is that even small changes in the background, such as the chest movements of a person breathing, or a person’s forearm exposed to biting mosquitoes in a cage, generate noise that may disturb the automated tracking. This problem can be addressed by either masking the moving object in the background or by creating a dynamic background with an algorithm that learns and updates over time [42]. Both approaches are complex and may affect tracking quality, sensitivity and computing power.

Background subtraction may be processed either in real-time or by analysing the recorded images post recording. Each approach has its pros and cons. Real-time processing requires less disk capacity by generating text files containing only the *x*-, *y*- and *z*-coordinates with a time stamp and even allows for using the animal’s position to trigger context-dependent stimuli in the experiment [43]. The downside is that additional information, such as the actual positions of other objects in the frame, is lost and must be reconstructed in other ways.

As already emphasised by Dickerson et al. [10], the best choice for a video tracking setup depends on a well-defined research question. The research question determines which basic features to prioritise and what adaptations in the video tracking setup are required. For example, to track mosquitoes throughout an entire night, disk capacity becomes critical, favouring a video tracking system that implements background subtraction in real-time over one that relies on post-processing of images. As several mosquitoes may be present within the same space simultaneously, for example when observing swarming, a video tracking system that can track multiple individuals is needed.

### What additional requirements are needed to track mosquitoes outside the controlled laboratory environment?

While a video tracking setup can be built on delicate high-tech equipment and optimised without major limitations for high performance in terms of spatial and temporal resolution in the laboratory, the field environment limits the deployment of such a video tracking system. A video tracking system deployed in the field needs to be robust, compact and versatile.

About three quarters of all mosquito species, including those most relevant for disease transmission, occur in the tropics and subtropics where the climate is humid and hot throughout the year [44]. Consequently, a video tracking system deployed in the field has to withstand those harsh climatic conditions. A more robust and compact system is also more likely to survive transportation to the field site and will be expected to be less prone to failure. Another consideration is that, in contrast to a laboratory setting, where parameters such as air-flow, climate and light conditions can be controlled and adjusted to the experimental setup, these parameters will follow natural fluctuations in a field environment. Moreover, the aim of studying behaviour in the field is to include the natural background in the experiment. Therefore, if the setup is too bulky, the video tracking may interfere with the environment and affect mosquito behaviour in a way that biases the interpretation of the results.

An intermediate step to study mosquito behaviour in the real world is to conduct experiments in semi-field systems, including large enclosed environments with screens [45] and experimental huts for product testing [46], before they are tested in large community trials. Insecticide-treated nets and IRS products are typically evaluated in experimental huts [47]. Experimental huts serve as a link between the laboratory and the field. They evaluate the products under a real use case scenario against wild mosquitoes and local weather conditions in structures made of local material, while the test procedures and the design of the huts remain consistent, providing some control over the experimental conditions. However, as the hut designs represent local architectures and are influenced by the researchers’ individual preferences, their design varies considerably between study sites [5]. The hut design may influence mosquito behaviour, affecting the outcomes of end point measurements (e.g. blood-feeding or mortality) [48], and each hut type poses new challenges for setting up an automated video tracking system. Therefore, the ideal video tracking system should not only be efficient but also versatile to match the different experimental hut conditions as well as in view of comparing mosquito behaviour across different hut types—or various types of behaviours (e.g. swarming, attraction to traps)—while using the same video tracking system.

Given the above considerations, not every automated video tracking system is equally well suited for measuring mosquito behaviour in the field, making the choice for the best fit challenging. To guide the selection of a suitable video tracking system, we reviewed the literature, and we compared and ranked the identified systems for their potential suitability in the field context taking into account the above considerations.

### Literature search

We searched Google Scholar (https://scholar.google.com) and PubMed (https://pubmed.ncbi.nlm.nih.gov) for literature describing automated video tracking systems that either have been used or could potentially be used for tracking free-flying mosquitoes. We considered both citations and patents up to 14 August 2024. The search string ‘mosquitoes AND flight AND video AND tracking’ generated 25 results in PubMed. In Google Scholar, the term ‘video tracking’ needed to be entered as a single term. The search string ‘mosquito AND flight AND video tracking’ generated 520 hits. We excluded 479 publications since they provided either no technical information about the tracking system used in the study or were off topic, cross references or duplicates. Once we had established a set of relevant systems, we did a second search iteration to find publications describing those systems, using the search string ‘insect OR mosquito AND flight AND video tracking AND “name of the system”’. If a publication reported an experimental setup identical with a previously described one, we also excluded the reference, and we only referred to the original publication.

From the selected publications, we then evaluated which are the most ‘established’ video tracking systems. We defined a system as being established if it (i) can track flying mosquitoes and (ii) is accessible to the wider research community as either (iia) a commercial product or (iib) an open-source software used by more than one research group.

The literature review summarises the most frequently used automated video tracking systems to analyse mosquito free-flight behaviour according to the above literature search and inclusion criteria. A detailed description of the created database can be found in Additional file 1.

### Similarities and differences in basic features among automated video tracking systems

We identified eight video tracking systems that met our inclusion criteria and have been deployed in a range of settings to study mosquito behaviour (Table 1). Of those systems, three have already been deployed in (semi-)field settings, including ‘Braid’ [49], ‘EthoVision XT’ [20, 22] and ‘StreamPix’ [13–15, 19, 50, 51]. Some come as complete systems (e.g. EthoVision XT) or as software that can be combined with the hardware of choice and complemented with other software (e.g. StreamPix). Multiple other systems can reliably track mosquitoes; however, they did not meet our inclusion criteria of being established, for example, the acoustic-based tracking approach from Johnson et al. [52] or the system from Javed et al. that uses a Convolutional Neural Network to track multiple mosquitoes [53–55]. Some of those systems are summarised by Adjé et al. [56] and Javed et al. [57].

**Table 1:** Automated video tracking systems for tracking mosquitoes and previous use cases

System	Accessibility criteria	Availability	Integrated software for data analysis	Use cases
EthoVision XT	Commercial product	Noldus, Wageningen, The Netherlands	Yes	Tunnel assay [11, 58–61] Indoor flight arena [62–64] Experimental hut: trap study [24, 65] and house entry [20, 22] Other [35, 66]
Braid^a^	Open source	Caltec University, Pasadena, California, USA	Partially [49]	Tunnel assay [28, 67, 68] Flight arena [23, 69, 70]
Motus	Open source	Vicon/ Contemplas, Kempten, Germany	No	Tunnel assay [9, 60] Flight arena [71]
Noldus Media Recorder	Commercial product	Noldus, Wageningen, The Netherlands	Yes	Lab: bed net [37, 72]
OpenCV	Open source	Apache 2-licensed^b^, USA	No	Flight arena [33, 73, 74] Outdoor video [75]
Photonic Fence	Commercial product	Photonic Sentry, Bellevue, Washington, USA	No	Laser induced killing [76–78]
StreamPix	Open access software	NorPix, Montreal, Canada/Liverpool School of Tropical Medicine, Liverpool, UK/University of Warwick, Coventry, UK	Yes	Lab: bed net [79–82] Experimental hut: bed net [17, 19, 50, 51] Field: swarming behaviour [13–15]
Trackit3D	Commercial product	SciTracks, Bertschikon, Switzerland	Partially	Tunnel assay [18, 29, 31, 32, 34, 83, 84] Indoor flight arena [43, 84–93] Other [42, 94]

The table lists automated video tracking systems that are accessible either commercially or as open-source software used by multiple research groups

^a^Braid is the successor of Flydra; therefore, publications describing the Flydra video tracking system are integrated into the description about Braid

^b^An Apache 2-license is a permissive license. The end user is allowed to use the software for any purpose, therefore, also to modify and distribute it

**Fig. 1:**
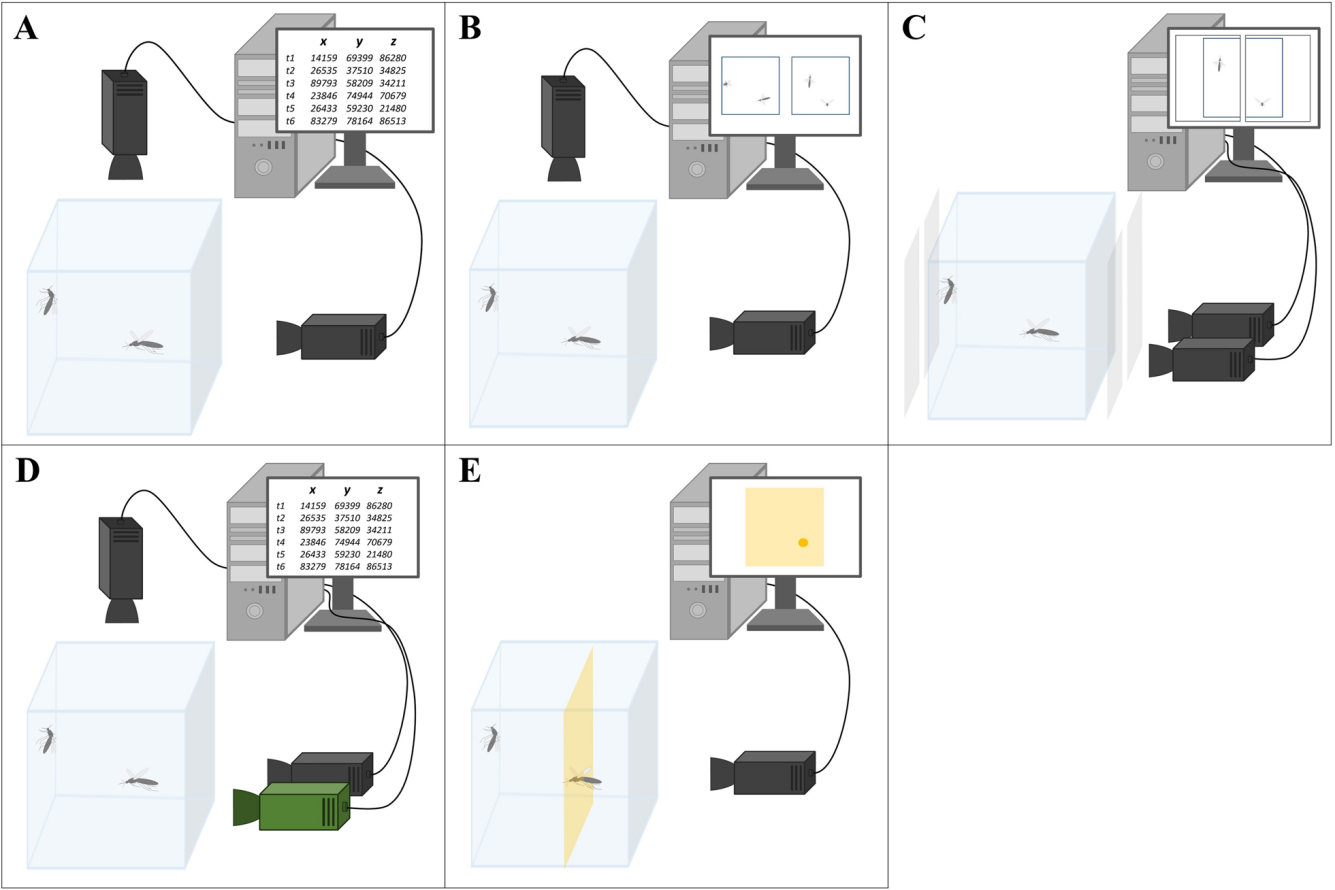
Differences in experimental setups and data processing among automated video tracking systems for tracking mosquitoes. The panels show the principle arrangements of the various setups and how data are processed in the initial step of recording for the identified automated video tracking systems. **A** Braid, EthoVision XT, OpenCV and Trackit3D work with two cameras that have a real-time analysis algorithm, so that the *x*-, *y*- and *z*–coordinates are instantly extracted. **B** Motus records videos with two cameras and post-processes them to extract the 3D positional data. An earlier version of StreamPix also worked along the same principles. **C** The setup using StreamPix has two cameras next to each other to cover a larger area and two pairs of Fresnel lenses with each camera to improve lighting. The video cameras record videos that are post-processed to generate 2D time-referenced position data. **D** The Photonic Fence consists of two cooperating tracking systems. The tracking system with two cameras does ‘coarse tracking’ of the mosquito, while the third camera does the ‘fine tracking’. **E** Noldus Media Recorder projects a laser sheet and, when a mosquito crosses the projected plane, detects the position based on where the object had deflected the laser beam

Four of the eight automated video tracking systems (i.e. Braid [49, 95], EthoVision XT [96], ‘OpenCV’ [73] and ‘Trackit3D’ [85]) work on the same principle with two or more cameras pointing at the observation arena, creating a field of overlap between them (Fig. 1A). If there are no spatial restrictions, for example due to bulky equipment or an unfavourable room setup, the cameras can be freely placed around the experimental arena to optimise tracking conditions, and then the system is calibrated to obtain accurate position data. To facilitate this process, Trackit3D has an inbuilt software function allowing for calibrating the system by the user once the cameras are put in place by moving a checkerboard across the cameras’ field of view [85].

A light source, generally in the near-infrared spectrum allowing for tracking nocturnal mosquitoes, provides the cameras with enough light to produce a contrast between the mosquito and its background. We have identified three different lighting modes, including backlight lighting [18], lighting from the front (i.e. from the same side as the cameras) [86] or from the side [11], and retro-reflective lighting [79].

The most widely used video tracking system for the study of insect behaviour is EthoVision XT; we found 727 citations with our search terms, ‘insect’ OR ‘mosquito’, in Google Scholar. EthoVision XT is a general-purpose video tracking system that analyses movements and recognises distinct behavioural patterns, and it comes with an add-on software package, called ‘Track3D’, that allows for reconstructing either two- or three-dimensional (flight) paths from the recorded, stored images [11]. EthoVision XT has been deployed using a variety of setups to track different animals, including insects [96] and different mosquito species [11, 20, 60, 66, 97–99]. Wind tunnel experiments with two cameras for 3D tracking [61] revealed mosquitoes exposed to sub-lethal levels of pyrethroids showing a reduced time of activation to flight and flight direction in host-seeking behaviour [60]. Additional flight tunnel studies found—in contrast to the general notion—no evidence of the pyrethroid deltamethrin being repellent at close range [97]. In another tunnel assay, video tracking with EthoVision XT confirmed previous findings of CO_2_ being a synergist—but not a host-seeking cue on its own—for anthropophagic mosquitoes [61]. Intriguingly in the context of the present review, EthoVision XT was also deployed in the semi-field context to track mosquito flight behaviour around eave tubes [22] and around regular eves in experimental hut studies [20].

An alternative system to EthoVision XT that is also commercially available and has been broadly deployed for behavioural studies is Trackit3D. Trackit3D has been used to study the behaviour of different arthropods, including mosquitoes [18, 84, 91, 92], flies [29, 31, 32] and spiders [94], across a range of experimental setups from small cage experiments to larger room settings [87]. Müller and Robert [86] combined Trackit3D with a custom-made LabVIEW (National Instruments, Austin, Texas, USA) user interface to control the artificial call of a field cricket while tracking the flight trajectory of its acoustic parasite, *Ormia ochracea*, as it homed in on the loudspeaker. The software turned off the loudspeaker that was playing the cricket song at defined distances away from it. The experiment revealed the fly was capable of memorising the position of the speaker away from it up to a distance of 1.5 m.

Yet another video tracking system that records positional data in real-time is built into the Photonic Fence. The Photonic Fence detects passing mosquitoes in flight and kills them with a laser pulse [78]. The underlying video tracking system is based on the same principle as the real-time tracking systems described above, while it is separated into a ‘coarse tracking’ and a ‘fine tracking’ unit (Fig. 1D). The coarse tracking detects and tracks the insect in 3D and transfers its estimated position to the fine tracking system. The fine tracking system consists of a high-speed camera that then precisely tracks the insect’s position in a plane. The laser module is then co-aligned with the fine tracking and shoots a lethal laser pulse at the flying mosquito. The two tracking systems do work partly independently from each other and may also only be used for tracking mosquitoes in 3D. Keller et al. [77] showed that the system could track mosquitoes up to > 30 m in an experimental setup with back lighting.

In contrast to commercial systems, EthoVision XT and Trackit3D, the open-source software packages Braid and OpenCV require a good level of technical software and hardware expertise for their setup. OpenCV [33] provides a toolbox that can be combined with other software to build a customised tracking system. For example, ‘StreamPix’ has been combined with OpenCV to enhance noise filtering [79]. Consequently, almost every publication describes a new variation of OpenCV so that it comes across in the literature as a very heterogeneous system, while, in fact, the basic setup remains the same.

‘Braid’, the other open-source system, is the successor of ‘Flydra’ [100] and is based on the software ‘Motmot’ [95]. The system tracks mosquitoes at high speed and, therefore, may be particularly useful for exploring flight manoeuvres in more detail with a temporal resolution of up to 12,000 frames per second (fps). Cribellier et al. [23] used Braid to study the escape behaviour of *Aedes* and *Anopheles* mosquitoes when they were about to be struck by a mechanical swatter. They found that the mosquitoes modified their flight behaviour depending on light intensity, maximising their escape behaviour during their natural diel activity pattern.

In contrast to the four video tracking systems described above that process positional data in real-time, ‘Motus’ stores recorded videos for post-processing. Nonetheless, apart from the data processing, the setups deployed with Motus were similar to the ones described above (Fig. 1B). Motus has been used early on to describe important behavioural traits and responses of host-seeking *Aedes aegypti* females and revealed how they are instantly sensitised to human skin odours in the presence of CO_2_ [9]. While Motus has not been used much otherwise, and the technical aspects of the system are only sparsely described in the literature, an interesting feature of Motus is its built-in software functions and algorithms. They can calculate the 3D coordinates from images recorded by other systems and even allow for reconstructing missing coordinates [71]. Making use of this functionality, Cohnstaedt et al. [60] combined Motus with EthoVision XT to digitise tracks where the mosquito had been missed in the first place.

Another software that implements background subtraction of stored image files and has been combined with various setups is StreamPix. StreamPix was first used by Butail et al. [15] to track swarming mosquitoes in 3D using a set of paired cameras to produce a stereoscopic image (Fig. 1B). Later, Parker et al. [17, 50, 51] deployed the software to measure and classify the behaviour of host-seeking *Anopheles gambiae* around ITNs in both a laboratory and a semi-field setup. The mosquitoes’ flight paths were recorded and reconstructed in 2D, while the tracking area around the ITN was increased by placing a pair of cameras in a parallel configuration on either end of one of the bed net’s side panels (Fig. 1C). To improve the image quality of the flying mosquitoes, the setup worked with back-lit imaging technique, in which the mosquitoes appeared as shadows. The shadows were produced by having the light sources placed behind the net and the cameras being mounted at the opposite side of the net. On each end of the net, a 1.4-m^2^-sized Fresnel lens was placed between the light source and the back of the net as well as between the front of the net and the camera, whereby for each light source the pair of Fresnel lenses and the corresponding camera were aligned coaxially to each other (Fig. 1C). To track mosquitoes in 3D with only one camera, Voloshin et al. [79] described an approach using StreamPix with a retro-reflective lighting system that creates a shadow of the mosquito that enables triangulation. However, to our knowledge, no study has been published that used this configuration to track mosquito flight paths in 3D.

A unique approach is taken by the ‘Noldus Media Recorder’ (Fig. 1E). Noldus Media Recorder does not record the object per se. Instead, a laser sheet is projected onto the area of interest, such as the surface of a bed net, and when a mosquito crosses that laser sheet, the light bouncing off the mosquito is recorded by a camera. Therefore, the position of the mosquito can only be determined across that two-dimensional plain. Including another laser colour, additional laser illumination sheets may be projected allowing for recording positions on another plane in parallel [72]. However, a mosquito outside these laser planes is invisible for the camera. While this setup leaves little room for tracking complete flight paths of mosquitoes, the system has proven suitable to detect mosquitoes passing through holes in a bed net [37, 72].

### Comparison of video tracking systems to track mosquitoes in the laboratory

To assess the performance of a tracking system in the laboratory setting, we focused on its actual tracking performance, since we assumed the laboratory environment as such does not impose technical limitations (e.g. power supply and sufficient disk capacity are available). Specifically, we evaluated six basic features, including (i) the ability of reconstructing flight trajectories in 3D, (ii) the light range in which the system can operate, (iii) the consistency in its implementation across different experimental scenarios, (iv) the size of the tracking volume and (v) the spatial and (vi) temporal resolution (Table 2). For each feature and video tracking system, we assigned a score between 1 and 3 with ‘1’ corresponding to low or poor performance and a ‘3’ meaning high or good performance. The detailed list corresponding to the assessments in Table 2 can be found in the Additional file 1, Table S1. We then added up the individual scores for each of the eight tracking systems while giving certain features more weight than others (Additional file 2, Table S4).

**Table 2:** Assessment of basic features of automated video tracking systems to track mosquitoes

System	Cameras (*n*)	Light source	Consistency	Tracking volume^a^	Spatial resolution (px)	Temporal resolution^b^
EthoVision XT	2 – 3	NIR	High	Large	1920 × 1080	Low
Braid	2 – 3	NIR	High	Large	1024 × 1024	High
Motus	2 – 3	NIR	Medium	Medium	–	Low
Noldus recorder	2 – 3	Laser	High	–^c^	–	Low
OpenCV	2 – 3	NIR	Low	Small	1280 × 1024	Medium
Photonic Fence^d^	2 + 1	NIR	High	Small	512 × 512	High
StreamPix	2 – 3	NIR	High	Large	4096 × 3072	Medium
Trackit3D	2 – 3	NIR	High	Large	768 × 576	Medium

^a^Small: ≤ 0.125 m^3^; medium: 0.125 – 1 m^3^; large: > 1 m^3^

^b^Low: ≤ 100 frames per second (fps); medium: 100–1000 fps; high: > 1000 fps; 1 fps = 1 Hz

^c^The tracking volume of ‘Noldus Media Recorder’ is not included as the tracking was exclusively in 2D (i.e. not a volume): the size of the tracking area was 30 cm × 60 cm[37]

^d^For the scoring, the specifications of the coarse (NIR) tracking element were considered, as this component characterises the free-flying behaviour of a mosquito in a room. In contrast, the high temporal resolution of the fine tracking aims to localise the mosquito very precisely rather than to characterise its flight path

Except for the Noldus Media Recorder, all systems are capable of 3D tracking, requiring at least two cameras to generate the stereoscopic information. Nevertheless, EthoVision XT and StreamPix have occasionally been used in 2D mode for tracking mosquitoes [17, 19, 51, 66, 82]. While the number of cameras per se is not central, using more cameras can increase the volume in which mosquitoes are tracked without compromising spatial resolution [69]. At the same time, more cameras increase the complexity of a setup and the cost for the extra equipment. For similar reasons, Angarita-Jaimes et al. [50] developed a 2D device for tracking mosquitoes around an ITN in combination with Fresnel lenses (Fig. 1C) with the argument that the device can track mosquitoes over larger volumes as otherwise multiple pairs of cameras would be required to track mosquitoes around an ITN in 3D. For 3D video tracking, the cameras’ fields of view have to overlap while the stereo information is most accurate if the cameras are positioned at an angle of 90° to each other [49]. However, due to spatial and practical constraints, the angle may also be more acute or obtuse, resulting in higher triangulation errors and therefore less accurate estimates of the mosquitoes’ 3D position.

The visible light spectrum for humans ranges from 280 nm to around 700 nm. Infrared (IR) light covers the spectrum with longer wavelengths from 700 nm to 1 mm. The range of shorter wavelengths within the IR spectrum, extending up to the beginning of visible light, is referred to as near-infrared (NIR) light [101]. To track nocturnal *Anopheles* mosquitoes, tracking needs to be done outside the visible light range [38]. Therefore, most systems use NIR-sensitive cameras and illuminate the flight arena with corresponding NIR light as most insects are blind in that spectrum, including mosquitoes [39]. Nevertheless, it is important that the light emitted from the NIR source is actually in the NIR band and does not radiate too much into the visible red light range. Particularly, short wavelengths close to the lower NIR range between 590–660 nm overlap with the hue of human skin and therefore become visible to mosquitoes. In fact, these wavelengths elicit a strong attractive response in mosquitoes [28]. Using NIR for lighting is not only desirable for nocturnal mosquitoes but also useful to study diurnal species such as *Aedes albopictus*. Together with an optical band-pass filter, NIR lighting can improve tracking even in bright light as contrasts are improved and reflections minimised.

All identified systems use a NIR light source and respective cameras to track mosquito flight behaviour, except for the Noldus Media Recorder, which operates with a laser beam instead [37]. It is unclear whether and how the laser would influence mosquito behaviour. The NIR wavelengths used in the studies depend on the camera hardware and ranged from a peak wavelength at 840 nm [84] to 940 nm [9]. The most frequently used NIR wavelength (mode) was 850 nm [17, 19, 34, 77, 79, 83, 88]. In addition to the seemingly sufficient NIR lighting, some studies complemented the IR lighting with light in the visible range [9, 58], although the reasons for that have not been mentioned. We can see three possible explanations for adding visible to NIR lighting. First, visible light could help the insect to orientate better, and a minimum amount of visible light is actually needed for the insect to initiate behaviour [39]. Second, it might mimic a more natural light source [84]; third, additional light in the visible range may simply be unavoidable, such as light emitted from displays of laboratory equipment or computers.

For the sake of data analysis, reproducibility of results and ease of use, the process of setting up and operating a tracking system should remain as consistent as possible across experiments and settings. Yet, consistency does not mean the opposite of flexibility. Here, ‘flexibility’ describes the possibility to adapt a system to different scenarios and settings. That is how versatile a system is, while ‘consistency’ relates to the process of setting up a system. We investigated how the systems were set up, and we considered a system to be more ‘consistent’ the less variation we found in the setup process across studies using the same system. Among the eight systems, we found two that were less consistent, including OpenCV and Motus. Motus was a bit more consistent than OpenCV, albeit this might be linked to the lower number of publications available. The lower consistency of OpenCV and Motus does not come as a surprise, since they are both open-source and provide a toolbox [33, 71] rather than a single-purpose kit.

As mosquitoes are flying insects, they need a 3D space to show their natural behaviour. Therefore, the volume in which mosquitoes can be tracked by an automated video system may represent a limitation for certain studies. To address this, we tried to assess the volume in which a mosquito can be reliably tracked by taking into account the system’s spatial resolution. However, the spatial resolution of the systems has not been mentioned in the literature. Instead, some studies report the total number of pixels (px) per frame [14, 15, 23, 74, 77, 102]; yet, without knowing the dimension of the image, these numbers do not provide information about the spatial resolution [103]. Nevertheless, we report the number of pixels in Table 2, but we did not consider this parameter for our evaluation.

Another aspect is that the tracking volumes described in the literature naturally depend on the dimensions of the flight arenas and on where the cameras were placed; therefore, they do not necessarily indicate the limits of a given system. Moreover, the tracking volume can also be increased by extending a setup with additional cameras. Finally, the volume in which mosquitoes can be tracked (i.e. the distance away from the cameras) essentially depends on the contrast between the mosquitoes and the background. Approaches have been discussed in the literature regarding the use of StreamPix to improve illumination by adding Fresnel lenses [50] and implementing diffuse retro-reflective imaging [79] to improve contrast.

In addition to the spatial resolution, the temporal resolution or frame rate, measured in fps, needs to be high enough to sufficiently resolve mosquito movement for a given research question. The systems reviewed used frame rates ranging from 0.03 fps (OpenCV) [104] to 9000 fps (Braid) [67]. A temporal resolution of 50 fps is sufficient to track reliably free-flying mosquitoes [70]. In contrast, resolving the detailed mechanics of flight manoeuvres, such as wing beat motions or sudden escape behaviours, requires a frame rate up to 24,000 fps [70]. Braid [23] and Photonic Fence are both capable of high-speed recording with over 1000 fps [77, 100], while the reviewed publications reported < 100 fps for Noldus Media Recorder, Motus and EthoVision XT [24, 71, 72], making them more suitable for tracking flight paths than for resolving fine details of flight mechanics.

### Comparison of automated video tracking systems to track mosquitoes in the field

In addition to its actual tracking performance, an automated video system needs to meet other requirements to be suitable for the field setting. We considered the following five features as particularly important: (i) how portable and (ii) flexible a system is, (iii) data storage requirements, (iv) the size of equipment and (v) the computer algorithm’s ability to track multiple mosquitoes simultaneously.

For an automated video tracking system to be suitable for use in the field, it should be compact, easy to (re-)assemble and versatile so that it can be easily set up in a new environment without the need, for example, for special means of transportation or building new structures. To assess the video tracking system, we captured these features with the terms ‘portability’, ‘flexibility’ and ‘equipment size’ for each of the eight identified video tracking systems (Table 3). Although it becomes increasingly easier to store large data volumes, disk capacity might still be a limitation for video tracking and data analysis in the field context as mosquito behaviour would ideally be studied over several hours such as overnight in an experimental hut. Finally, the system should be able to track multiple mosquitoes simultaneously as more than one mosquito may enter the flight arena.

**Table 3:** Assessment of basic features of automated video systems to track mosquitoes in the field

System	Portability^a^	Data storage size^b^	Flexibility^c^	Equipment size^d^	Multiple mosquitoes
EthoVision XT	Medium	Small	High	Small	Yes
Braid	Good	Small	Medium	Small	Yes
Motus	Good	Large	Medium	Small	Yes
Noldus recorder	Good	Large	Low	Small	Yes
OpenCV	Good	Small	Medium	Small	Yes
Photonic fence	Medium	Small	Low	Medium	Yes
StreamPix	Medium	Large	High	Large	Yes
Trackit3D	Good	Small	High	Small	Yes

^a^Good: equipment can be disassembled and reassembled without special expertise or packaging material; medium: requires expert knowledge and specialised material or equipment for the system to be moved from one place to another; poor: system can hardly be moved; yet, none of the reviewed systems were in this category

^b^Small: real-time tracking producing simple text files in the range of a few hundred kBs per 10 min; large: offline image analysis by which entire videos are required to be stored on a hard drive, producing up to several TBs of data for longer sessions (e.g. tracking over an entire night)

^c^Low: only one type of experiment has previously been done; medium: two different types of experiment have previously been done; high: three or more different experiments have previously been done

^d^Small: beyond cameras, lamps and computer, no specialised equipment needed; medium: additional equipment needed; large: bulky additional equipment required such as Fresnel lenses

**Fig. 2:**
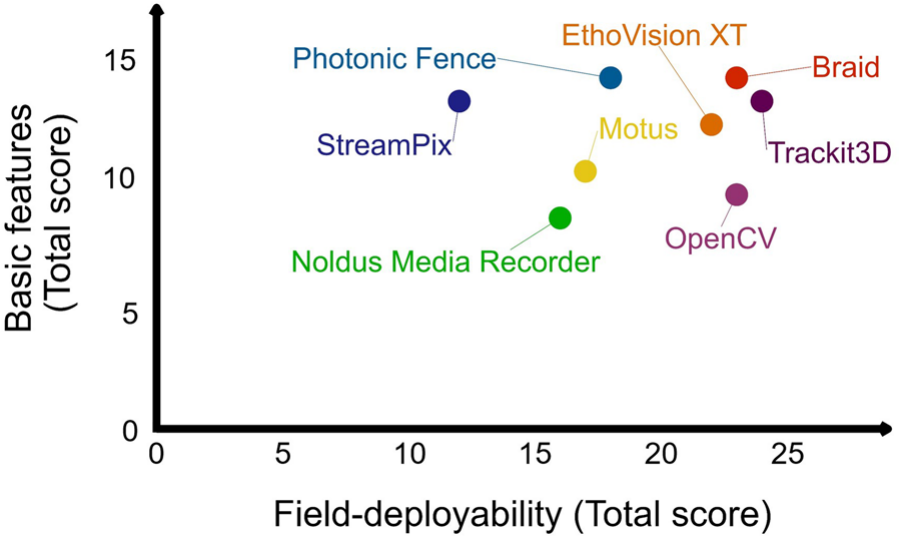
Suitability of automated video tracking systems to track mosquitoes in the field. Each point shows the results for one of eight automated video tracking systems to record mosquito flight behaviour in the field context, comparing the total score for its basic features against its total score for field deployability. The total scores are the sum of individual scores for different parameters (Tables 2 and 3). An ideal system has a high total score for both the basic features and its field deployability (i.e. features in the top right corner of the graph)

Similarly to the laboratory evaluation, where we looked at how well a system performs regarding the basic features, we assessed each system's suitability for use in the field setting, termed here as ‘field deployability’. Field deployability is a summary score based on the evaluation of each of the five features above. As for the basic features, we gave each system and feature a score between 1 and 3 (Table 3 and Additional file 1: Table S2) and then summed up the individual scores to compute a total score. We then compared the total score for field deployability with the total score for the basic features (Fig. 2). Based on our scoring and selection criteria, we identified Braid, EthoVision XT and Trackit3D to be the best performing systems. They show high total scores for both the basic features and field deployability. Braid, EthoVision XT and Trackit3D are well adaptable to various settings and require minimal storage capacity.

A detailed list of additional information such as different insects tracked can be found in the Supplementary (Additional file 1: Table S3).

How ‘portable’ a system is depends primarily on the size of the equipment and how complex the process is to set it up including its calibration in a new setting. A setup that seems rather bulky is the one based on the open source software StreamPix, which has been deployed for tracking *Anopheles* mosquitoes around ITNs in both the laboratory and an experimental hut in Tanzania [17, 51]. Two sets of two cameras were combined with each camera paired with an IR lamp for background lighting with two 1.0 m × 1.4 m Fresnel lenses, one in front of the camera and the other one between the IR lamp and the net, to collimate the light (Fig. 1C). As the camera system is not capable of 3D tracking and to allow for tracking on both the side and roof panel of the ITN, the net was custom-tailored with the roof lowered towards the side of the cameras. Together, this makes the system less portable and flexible. Although more flexible than the StreamPix setup above, we also deem EthoVision as less portable, since this is a commercial systems that depends on external support for its setup and calibration, providing little autonomy. Nevertheless, EthoVision has proved valuable for tracking mosquitoes in a field setting. Spitzen et al. [20] used EthoVision to observe house entry behaviour of *Anopheles* mosquitoes in 3D in a semi-field setting. Another system that can track in 3D, yet appears to be less suitable for the field setting, is the Photonic Fence. The Photonic Fence is a rather sophisticated system that depends on the interplay between two laser systems for coarse and fine tracking; thus, we expect this system to be more challenging to set up and maintain under field conditions. In addition, while both StreamPix and EthoVision have delivered valuable results on mosquito behaviour under semi-field conditions, we have not yet come across a study that presents semi-field or field data generated with the Photonic Fence.

Besides the portability, another limitation of tracking mosquitoes in a field setting can be access to computational power and sufficient disk capacity. Therefore, the required disk capacity should not quickly exceed the hardware’s limits. For example, automated video tracking in combination with StreamPix and two cameras generates about 2 TB of video files per hour [79]. Storage limitation becomes especially critical for field studies that would ideally track mosquitoes overnight such as in experimental huts. This might explain why Parker et al. [51] restricted their video recordings to 3.5 h per session and opted to release laboratory-reared mosquitoes instead of waiting for wild mosquitoes to enter the hut. Like StreamPix, Noldus Media Recorder stores video files on a disk first before they are processed with the software Noldus EthoVision [37, 72], and for Motus images can only be digitised offline [8]. The other systems (i.e. Braid, Photonic Fence and Trackit3D) allow for real-time analysis of the acquired images and instantly write the *x*-, *y*- and *z*-coordinates to the disk. In addition to reducing storage size, real-time tracking allows for triggering other applications such as a sound generator as a function of the object’s position (see description of Trackit3D above). The disadvantage of not storing video files is that one cannot directly relate the mosquito’s position to its surroundings. To reconcile the advantages of both approaches, real-time tracking may be combined with recording videos at a lower resolution, a strategy that has been implemented by Fatou and Müller [18] while tracking *Anopheles gambiae* s.s. around net samples﻿.

As multiple mosquitoes may appear in front of the cameras at the same time in a field setting, and since we might want to assign trajectories to each individual mosquito for the purpose of analysis, the software faces the problem that it needs to differentiate between individuals. All eight systems reviewed here can track multiple mosquitoes simultaneously from a few mosquitoes in the single digits [9, 67, 71, 75, 84, 91] up to hundreds of mosquitoes [20, 22, 33, 37, 65, 78, 102]. The limit in the number of mosquitoes being tracked simultaneously has, however, not been mentioned in the literature, and in the laboratory studies the authors did not always distinguish between how many mosquitoes were released and how many were actually tracked concurrently [20, 33]. Similarly, the field studies report the total number of mosquitoes present rather than the number of mosquitoes actually tracked at the same time [13–15, 17, 19, 20, 22, 24, 37, 50, 51, 65, 75].

Other aspects may influence the choice of a suitable system that we did not consider in our evaluation, as they have neither been reported in the literature nor were they available from other sources or our own experience. Conceivably, two additional aspects that may influence the choice of one system over another are its user-friendliness and price tag.

Some systems are likely more user-friendly than others; however, this parameter is difficult to quantify and may also depend on personal preferences. Open-source software typically offers greater flexibility but requires comprehensive skills in programming machine learning and computer vision algorithms [10], which can make it less accessible to the broader research community. In contrast, commercially available systems, while offering less flexibility, are likely designed for ease of use and include customer support.

The cost of purchasing and servicing a commercial system, as well as equipment expenses, can be important factors in selecting a system. Unfortunately, we were unable to retrieve comprehensive cost information across all video tracking systems from the literature or other sources to include in our evaluation. Costs also vary depending on the experimental setup and the specific equipment and software features required. Based on our experience, the estimated cost of a setup using open-source software—including a desktop computer, two mid-range NIR video cameras with fittings (e.g. lenses, filters and cables), IR lamps and camera rigs—is about USD 10,000–15,000. In contrast, a commercial product with a software license may cost many times more.

## Conclusions

While all the systems reviewed have provided invaluable insights into mosquito behaviour that would otherwise be difficult to investigate, Braid, EthoVision XT and Trackit3D stand out as the most promising video tracking systems for field deployment. Their good performance in tracking flying insects in 3D, along with properties that enhance their suitability for use, make them particularly advantageous. However, the ultimate choice for the most suitable tracking system will depend on several factors, including the specific research question, budget constraints and the desired features critical for the study’s objectives. Prioritising one feature may necessitate compromises in others, making it essential to carefully evaluate the required properties. Therefore, no single video tracking system can be considered universally superior.

Autonomous video tracking of mosquito flight paths will undoubtedly grow in relevance, since it has already revealed previously elusive behaviours and continues to gain recognition for its critical role in understanding mosquito behaviour [57]. Tracking mosquito flight and other behaviours remains a challenging task, requiring a lot of technical expertise, especially for its application in the field context. However, promising advancements in video tracking technology are emerging, driven by developments in computer technology, including artificial intelligence and deep-learning algorithms [53–55]. Similarly, data analysis will likely become increasingly automated and efficient [10]. Together, these innovations may expand the potential for automated video tracking of mosquito behaviour, even in more complex environments such as the semi-field setting.

## Supplementary Information

Additional file 1

Additional file 2

Additional file 3

## Data Availability

Data are provided within the manuscript or supplementary information files. A list of all publications included in the review can be found in 'Additional file 3, Table S5'.

## Abbreviations

2D: Two-dimensional or two dimensions
3D: Three-dimensional or three dimensions
ACT: Artemisinin-based combination therapy
fps: Frames per second
IRS: Indoor residual spraying
ITN: Insecticide-treated bed net
NIR: Near-infrared
px: Pixel
Swiss TPH: Swiss Tropical and Public Health Institute
WHO: World Health Organization
