# Supplementary material for: Revealing complex mosquito behaviour: a review of current automated video tracking systems suitable for tracking mosquitoes in the field
Source: Parasit Vectors. 2025 Feb 21;18:66. doi: 10.1186/s13071-025-06666-6 (PMC11846416; doi:10.1186/s13071-025-06666-6)
Supplement: Supplementary file 2 — Additional file 2 [file 13071_2025_6666_MOESM2_ESM.docx]

**Additional file 2: Table** **S4: Weight of the different basic features and field deployability of the revived video tracking systems**

| **Class** | **Characteristic** | **Weighting** |
| --- | --- | --- |
| Basic features | Number of cameras | 1 |
| Basic features | Lighting^1^ | 2 |
| Basic features | Consistency of the system^2^ | 2 |
| Basic features | Spatial resolution | 1 |
| Basic features | Temporal resolution | 2 |
| Field deployability | Portability^4^ | 2 |
| Field deployability | Data storage size | 3 |
| Field deployability | Flexibility^5^ | 1 |
| Field deployability | Equipment size | 2 |
| Field deployability | Multiple mosquitoes | 3 |

The type of lighting that is possible (qualitative factor) ^2^Consistency in the modifications reported across different studies using the same system. 3 Volume that is recorded. ^4^How good the system can be packed into a container and transported to the field site. ^5^Flexibility concerning different use cases, applications and camera placement.

As an example, Motus has a recording area of 150 cm x 50 cm x 50 cm what is considered as *medium performance* */ 2 points [1]*. ‘Tracking Volume’ has a weight of *3,* and therefore we added *6* points to the basic characteristics score of Motus. The final score should correlate with the potential performance of the system.

## References

1. Dekker T, Geier M, Carde RT. Carbon dioxide instantly sensitizes female yellow fever mosquitoes to human skin odours. J Exp Biol. 2005;208 Pt 15:2963-72; doi: 10.1242/jeb.01736. <https://www.ncbi.nlm.nih.gov/pubmed/16043601>.
