# Supplementary material for: Revealing complex mosquito behaviour: a review of current automated video tracking systems suitable for tracking mosquitoes in the field
Source: Parasit Vectors. 2025 Feb 21;18:66. doi: 10.1186/s13071-025-06666-6 (PMC11846416; doi:10.1186/s13071-025-06666-6)
Supplement: Supplementary file 3 — Additional file 3 [file 13071_2025_6666_MOESM3_ESM.docx]

**Additional file 3: Table S5: References**

| System | References | Total |
| --- | --- | --- |
| EthoVision XT | [1, 2, 3, 4, 5, 6, 7, 8, 9, 10, 11, 12, 13, 14] | 14 |
| Braid | [15, 16, 17, 18, 19, 20, 21, 22] | 8 |
| Motus | [5, 23, 24] | 3 |
| Noldus recorder | [25, 26] | 2 |
| OpenCV | [27, 28, 29, 30, 31, 32] | 6+n^1^ |
| Photonic fence | [33, 34, 35] | 3 |
| StreamPix | [36, 37, 38, 39, 40, 41, 42, 43, 44, 45, 46] | 11 |
| Trackit3D | [47, 48, 49, 50, 51, 52, 53, 54, 55, 56, 57, 58, 59, 60, 61, 62, 63, 64, 65] | 19 |

[27] is a PhD thesis. Therefore, it contains numerous different publications such as [66].

## References

1. Costa AA, Gonzalez PV, Harburguer LV, Masuh HM. A rapid method for screening mosquito repellents on Anopheles pseudopunctipennis and Aedes aegypti. Parasitol Res. 2022;121 9:2713-23; doi: 10.1007/s00436-022-07600-w. <https://www.ncbi.nlm.nih.gov/pubmed/35867157>.

2. Diop MM, Moiroux N, Chandre F, Martin-Herrou H, Milesi P, Boussari O, et al. Behavioral cost & overdominance in Anopheles gambiae. PLoS One. 2015;10 4:e0121755; doi: 10.1371/journal.pone.0121755. <https://www.ncbi.nlm.nih.gov/pubmed/25831058>.

3. Spitzen J, Koelewijn T, Mukabana WR, Takken W. Visualization of house-entry behaviour of malaria mosquitoes. Malar J. 2016;15:233; doi: 10.1186/s12936-016-1293-7. <https://www.ncbi.nlm.nih.gov/pubmed/27108961>.

4. Spitzen J, Spoor CW, Grieco F, ter Braak C, Beeuwkes J, van Brugge SP, et al. A 3D analysis of flight behavior of Anopheles gambiae sensu stricto malaria mosquitoes in response to human odor and heat. PLoS One. 2013;8 5:e62995; doi: 10.1371/journal.pone.0062995. <https://www.ncbi.nlm.nih.gov/pubmed/23658792>.

5. Cohnstaedt LW, Allan SA. Effects of sublethal pyrethroid exposure on the host-seeking behavior of female mosquitoes. journal of vectror ecology. 2011;36 2:395 - 403; doi: 10.1111/j.1948-7134.2011.00180.x.

6. Lacey ES, Carde RT. Activation, orientation and landing of female Culex quinquefasciatus in response to carbon dioxide and odour from human feet: 3-D flight analysis in a wind tunnel. Med Vet Entomol. 2011;25 1:94-103; doi: 10.1111/j.1365-2915.2010.00921.x. <https://www.ncbi.nlm.nih.gov/pubmed/21118282>.

7. Amos BA, Staunton KM, Ritchie SA, Carde RT. Attraction Versus Capture: Efficiency of BG-Sentinel Trap Under Semi-Field Conditions and Characterizing Response Behaviors for Female Aedes aegypti (Diptera: Culicidae). J Med Entomol. 2020;57 3:884-92; doi: 10.1093/jme/tjz243. <https://www.ncbi.nlm.nih.gov/pubmed/31977049>.

8. Batista EPA, Mapua SA, Ngowo H, Matowo NS, Melo EF, Paixao KS, et al. Videographic analysis of flight behaviours of host-seeking Anopheles arabiensis towards BG-Malaria trap. PLoS One. 2019;14 7:e0220563; doi: 10.1371/journal.pone.0220563. <https://www.ncbi.nlm.nih.gov/pubmed/31365584>.

9. Hinze A, Lantz J, Hill SR, Ignell R. Mosquito Host Seeking in 3D Using a Versatile Climate-Controlled Wind Tunnel System. Front Behav Neurosci. 2021;15:643693; doi: 10.3389/fnbeh.2021.643693. <https://www.ncbi.nlm.nih.gov/pubmed/33776664>.

10. Lee DH, Wright SE, Leskey TC. Impact of insecticide residue exposure on the invasive pest, Halyomorpha halys (Hemiptera: Pentatomidae): analysis of adult mobility. J Econ Entomol. 2013;106 1:150-8; doi: 10.1603/ec12265. <https://www.ncbi.nlm.nih.gov/pubmed/23448027>.

11. Martin T, Palix R, Kamal A, Deletre E, Bonafos R, Simon S, et al. A repellent net as a new technology to protect cabbage crops. J Econ Entomol. 2013;106 4:1699-706; doi: 10.1603/ec13004. <https://www.ncbi.nlm.nih.gov/pubmed/24020284>.

12. Sperling S, Cordel M, Gordon S, Knols BGJ, Rose1 A. Eave tubes for malaria control in Africa: Videographic observations of mosquito behaviour in Tanzania with a simple and rugged video surveillance system. MalariaWorld Journal. 2017;8 9.

13. Hug DOH, Kropf A, Amann MO, Koella JC, Verhulst NO. Unexpected behavioural adaptation of yellow fever mosquitoes in response to high temperatures. Scientific Reports. 2024;14 1:3659; doi: 10.1038/s41598-024-54374-5. <https://doi.org/10.1038/s41598-024-54374-5>.

14. Park Y-g, Lee YS, Sarker S, Ham EH, Lim UT. Attractiveness of four wavelengths of LED light: UV (385 nm), violet (405 nm), blue (450 nm), and red (660 nm) for seven species of natural enemies. Biological Control. 2023;179; doi: 10.1016/j.biocontrol.2023.105166.

15. Ahmed I, Faruque IA. High speed visual insect swarm tracker (Hi-VISTA) used to identify the effects of confinement on individual insect flight. Bioinspir Biomim. 2022;17 4; doi: 10.1088/1748-3190/ac6849. <https://www.ncbi.nlm.nih.gov/pubmed/35439741>.

16. Cribellier A, Straw AD, Spitzen J, Pieters RPM, van Leeuwen JL, Muijres FT. Diurnal and nocturnal mosquitoes escape looming threats using distinct flight strategies. Curr Biol. 2022;32 6:1232-46 e5; doi: 10.1016/j.cub.2022.01.036. <https://www.ncbi.nlm.nih.gov/pubmed/35134328>.

17. Maimon G, Straw AD, Dickinson MH. A simple vision-based algorithm for decision making in flying Drosophila. Curr Biol. 2008;18 6:464-70; doi: 10.1016/j.cub.2008.02.054. <https://www.ncbi.nlm.nih.gov/pubmed/18342508>.

18. Straw AD, Branson K, Neumann TR, Dickinson MH. Multi-camera real-time three-dimensional tracking of multiple flying animals. J R Soc Interface. 2011;8 56:395-409; doi: 10.1098/rsif.2010.0230. <https://www.ncbi.nlm.nih.gov/pubmed/20630879>.

19. Straw AD, Dickinson MH. Motmot, an open-source toolkit for realtime video acquisition and analysis. Source Code Biol Med. 2009;4:5; doi: 10.1186/1751-0473-4-5. <https://www.ncbi.nlm.nih.gov/pubmed/19624853>.

20. Straw AD, Pieters R, Muijres FT. Real-Time Tracking of Multiple Moving Mosquitoes. Cold Spring Harb Protoc. 2022; doi: 10.1101/pdb.prot107927. <https://www.ncbi.nlm.nih.gov/pubmed/36171067>.

21. Alonso San Alberto D, Rusch C, Zhan Y, Straw AD, Montell C, Riffell JA. The olfactory gating of visual preferences to human skin and visible spectra in mosquitoes. Nat Commun. 2022;13 1:555; doi: 10.1038/s41467-022-28195-x. <https://www.ncbi.nlm.nih.gov/pubmed/35121739>.

22. Muijres FT, Dickerson AK, Pieters R. Designing a Generic Videography Experiment for Studying Mosquito Behavior. Cold Spring Harb Protoc. 2022; doi: 10.1101/pdb.prot107926. <https://www.ncbi.nlm.nih.gov/pubmed/36167671>.

23. Cooperband MF, T. CR. Orientation of Culex mosquitoes to carbon dioxice-baited traps:flight manoeuvres and trapping efficiency. Med Vet Entomol. 2006;20:11-26; doi: 10.1111/j.1365-2915.2006.00613.x.

24. Dekker T, Geier M, Carde RT. Carbon dioxide instantly sensitizes female yellow fever mosquitoes to human skin odours. J Exp Biol. 2005;208 Pt 15:2963-72; doi: 10.1242/jeb.01736. <https://www.ncbi.nlm.nih.gov/pubmed/16043601>.

25. Sutcliffe J, Ji X, Yin S. How many holes is too many? A prototype tool for estimating mosquito entry risk into damaged bed nets. Malar J. 2017;16 1:304; doi: 10.1186/s12936-017-1951-4. <https://www.ncbi.nlm.nih.gov/pubmed/28764726>.

26. Sutcliffe JF, Yin S. Effects of indoor air movement and ambient temperature on mosquito (Anopheles gambiae) behaviour around bed nets: implications for malaria prevention initiatives. Malar J. 2021;20 1:427; doi: 10.1186/s12936-021-03957-y. <https://www.ncbi.nlm.nih.gov/pubmed/34717652>.

27. Kimura T: **Development of automatic tracking mehtods for the analysis of animal behaviors**. *A dissertation submitted in partial fulfillment of the requirements for the degree of Doctor of Engineering.* Hongo Japan: University of Hyogo, Japan; 2017.

28. Khan B, Gaburro J, Hanoun S, Duchemin J-B, Nahavandi S, Bhatti A. Activity and Flight Trajectory Monitoring of Mosquito Colonies for Automated Behaviour Analysis. Neural Information Processing; 2015. p. 548-55.

29. Poh AH, Moghavvemi M, Leong CS, Lau YL, Safdari Ghandari A, Apau A, et al. Collective behavior quantification on human odor effects against female Aedes aegypti mosquitoes-Open source development. PLoS One. 2017;12 2:e0171555; doi: 10.1371/journal.pone.0171555. <https://www.ncbi.nlm.nih.gov/pubmed/28152031>.

30. Islam MS, Faruque IA. Experimental identification of individual insect visual tracking delays in free flight and their effects on visual swarm patterns. 2022; doi: 10.1101/2022.04.06.487367.

31. Fennell JT, Wilby A, Sobeih W, Paul ND. New understanding of the direct effects of spectral balance on behaviour in Myzus persicae. J Insect Physiol. 2020;126:104096; doi: 10.1016/j.jinsphys.2020.104096. <https://www.ncbi.nlm.nih.gov/pubmed/32800776>.

32. Ratnayake MN, Dyer AG, Dorin A. Tracking individual honeybees among wildflower clusters with computer vision-facilitated pollinator monitoring. PLoS One. 2021;16 2:e0239504; doi: 10.1371/journal.pone.0239504. <https://www.ncbi.nlm.nih.gov/pubmed/33571210>.

33. Keller MD, Leahy DJ, Norton BJ, Johanson T, Mullen ER, Marvit M, et al. Laser induced mortality of Anopheles stephensi mosquitoes. Sci Rep. 2016;6:20936; doi: 10.1038/srep20936. <https://www.ncbi.nlm.nih.gov/pubmed/26887786>.

34. Mullen ER, Rutschman P, Pegram N, Patt JM, Adamczyk JJ, Johanson. Laser system for identification, tracking, and control of flying insects. Opt Express. 2016;24 11:11828-38; doi: 10.1364/OE.24.011828. <https://www.ncbi.nlm.nih.gov/pubmed/27410106>.

35. Keller MD, Norton BJ, Farrar DJ, Rutschman P, Marvit M, Makagon A. Optical tracking and laser-induced mortality of insects during flight. Sci Rep. 2020;10 1:14795; doi: 10.1038/s41598-020-71824-y. <https://www.ncbi.nlm.nih.gov/pubmed/32908169>.

36. Butail S, Manoukis N, Diallo M, Yaro AS, Dao A, Traore SF, et al. 3D tracking of mating events in wild swarms of the malaria mosquito Anopheles gambiae. Annu Int Conf IEEE Eng Med Biol Soc. 2011;2011:720-3; doi: 10.1109/IEMBS.2011.6090163. <https://www.ncbi.nlm.nih.gov/pubmed/22254411>.

37. Butail S, Manoukis N, Diallo M, Ribeiro JM, Lehmann T, Paley DA. Reconstructing the flight kinematics of swarming and mating in wild mosquitoes. J R Soc Interface. 2012;9 75:2624-38; doi: 10.1098/rsif.2012.0150. <https://www.ncbi.nlm.nih.gov/pubmed/22628212>.

38. Butail S, Manoukis NC, Diallo M, Ribeiro JM, Paley DA. The dance of male Anopheles gambiae in wild mating swarms. J Med Entomol. 2013;50 3:552-9; doi: 10.1603/me12251. <https://www.ncbi.nlm.nih.gov/pubmed/23802449>.

39. Parker JE, Angarita-Jaimes N, Abe M, Towers CE, Towers D, McCall PJ. Infrared video tracking of Anopheles gambiae at insecticide-treated bed nets reveals rapid decisive impact after brief localised net contact. Sci Rep. 2015;5:13392; doi: 10.1038/srep13392. <https://www.ncbi.nlm.nih.gov/pubmed/26323965>.

40. Angarita-Jaimes NC, Parker JE, Abe M, Mashauri F, Martine J, Towers CE, et al. A novel video-tracking system to quantify the behaviour of nocturnal mosquitoes attacking human hosts in the field. J R Soc Interface. 2016;13 117; doi: 10.1098/rsif.2015.0974. <https://www.ncbi.nlm.nih.gov/pubmed/27075002>.

41. Parker JEA, Angarita Jaimes NC, Gleave K, Mashauri F, Abe M, Martine J, et al. Host-seeking activity of a Tanzanian population of Anopheles arabiensis at an insecticide treated bed net. Malar J. 2017;16 1:270; doi: 10.1186/s12936-017-1909-6. <https://www.ncbi.nlm.nih.gov/pubmed/28676092>.

42. Murray GPD, Lissenden N, Jones J, Voloshin V, Toe KH, Sherrard-Smith E, et al. Barrier bednets target malaria vectors and expand the range of usable insecticides. Nat Microbiol. 2020;5 1:40-7; doi: 10.1038/s41564-019-0607-2. <https://www.ncbi.nlm.nih.gov/pubmed/31792426>.

43. Voloshin V, Kroner C, Seniya C, Murray GPD, Guy A, Towers CE, et al. Diffuse retro-reflective imaging for improved video tracking of mosquitoes at human baited bednets. R Soc Open Sci. 2020;7 5:191951; doi: 10.1098/rsos.191951. <https://www.ncbi.nlm.nih.gov/pubmed/32537200>.

44. Abbott AJ, Matope A, Jones J, Voloshin V, Towers CE, Towers D, et al. Insecticidal roof barriers mounted on untreated bed nets can be as effective against Anopheles gambiae as regular insecticide-treated bed nets. Sci Rep. 2023;13 1:22080; doi: 10.1038/s41598-023-48499-2.

45. Gleave K, Guy A, Mechan F, Emery M, Murphy A, Voloshin V, et al. Impacts of dual active-ingredient bed nets on the behavioural responses of pyrethroid resistant Anopheles gambiae determined by room-scale infrared video tracking. Malar J. 2023;22 1:132; doi: 10.1186/s12936-023-04548-9.

46. Parker JEA, Kakilla C, Nelwin K, Kroner C, Logan R, Ismail HM, et al. Video-tracked Anopheles arabiensis entry and exit behaviour at washed and damaged pyrethroid-treated bednets. Royal Society Open Science. 2024;11 5:231165; doi: doi:10.1098/rsos.231165 %U <https://royalsocietypublishing.org/doi/abs/10.1098/rsos.231165>.

47. Fry SN, Bichsel M, Müller P, Robert D. Tracking of flying insects using pan-tilt cameras. j Neurosci Methods. 2000;101.

48. Müller P, Robert D. A shot in the the dark: the silent quest of a free-flying phonotactic fly. J Exp Biol. 2001;204:1039–52; doi: 10.1242/jeb.204.6.1039.

49. Müller P, Robert D. Death comes suddenly to the unprepared: singing crickets, call fragmentation, and parasitoid flies. Behavioral Ecology. 2002;13 5:598–606; doi: 10.1093/beheco/13.5.598.

50. Fry SN, Muller P, Baumann HJ, Straw AD, Bichsel M, Robert D. Context-dependent stimulus presentation to freely moving animals in 3D. J Neurosci Methods. 2004;135 1-2:149-57; doi: 10.1016/j.jneumeth.2003.12.012. <https://www.ncbi.nlm.nih.gov/pubmed/15020099>.

51. Ribak G, Swallow JG. Free flight maneuvers of stalk-eyed flies: do eye-stalks affect aerial turning behavior? J Comp Physiol A Neuroethol Sens Neural Behav Physiol. 2007;193 10:1065-79; doi: 10.1007/s00359-007-0259-1. <https://www.ncbi.nlm.nih.gov/pubmed/17710410>.

52. Rouyar Al, Deisig N, Dupuy F, Limousin D, Wycke M-A, Renou M, et al. Unexpected plant odor responses in a moth pheromone system. Frontiers in Physiology. 2015;6; doi: 10.3389/fphys.2015.00148.

53. Dupuy F, Rouyar A, Deisig N, Bourgeois T, Limousin D, Wycke MA, et al. A Background of a Volatile Plant Compound Alters Neural and Behavioral Responses to the Sex Pheromone Blend in a Moth. Front Physiol. 2017;8:79; doi: 10.3389/fphys.2017.00079. <https://www.ncbi.nlm.nih.gov/pubmed/28239358>.

54. Budick SA, Dickinson MH. Free-flight responses of Drosophila melanogaster to attractive odors. J Exp Biol. 2006;209 Pt 15:3001-17; doi: 10.1242/jeb.02305. <https://www.ncbi.nlm.nih.gov/pubmed/16857884>.

55. Almbro M, Kullberg C. Impaired escape flight ability in butterflies due to low flight muscle ratio prior to hibernation. J Exp Biol. 2008;211 Pt 1:24-8; doi: 10.1242/jeb.008219. <https://www.ncbi.nlm.nih.gov/pubmed/18083728>.

56. Ings TC, Chittka L. Speed-accuracy tradeoffs and false alarms in bee responses to cryptic predators. Curr Biol. 2008;18 19:1520-4; doi: 10.1016/j.cub.2008.07.074. <https://www.ncbi.nlm.nih.gov/pubmed/18771920>.

57. McMeniman CJ, Corfas RA, Matthews BJ, Ritchie SA, Vosshall LB. Multimodal integration of carbon dioxide and other sensory cues drives mosquito attraction to humans. Cell. 2014;156 5:1060-71; doi: 10.1016/j.cell.2013.12.044. <https://www.ncbi.nlm.nih.gov/pubmed/24581501>.

58. Hawkes F, Gibson G. Seeing is believing: the nocturnal malarial mosquito Anopheles coluzzii responds to visual host-cues when odour indicates a host is nearby. Parasit Vectors. 2016;9 1:320; doi: 10.1186/s13071-016-1609-z. <https://www.ncbi.nlm.nih.gov/pubmed/27260254>.

59. Lihoreau M, Ings TC, Chittka L, Reynolds AM. Signatures of a globally optimal searching strategy in the three-dimensional foraging flights of bumblebees. Sci Rep. 2016;6:30401; doi: 10.1038/srep30401. <https://www.ncbi.nlm.nih.gov/pubmed/27459948>.

60. Houot B, Gigot V, Robichon A, Ferveur JF. Free flight odor tracking in Drosophila: Effect of wing chemosensors, sex and pheromonal gene regulation. Sci Rep. 2017;7:40221; doi: 10.1038/srep40221. <https://www.ncbi.nlm.nih.gov/pubmed/28067325>.

61. Houot B, Cazale-Debat L, Fraichard S, Everaerts C, Saxena N, Sane SP, et al. Gene Regulation and Species-Specific Evolution of Free Flight Odor Tracking in Drosophila. Mol Biol Evol. 2018;35 1:3-15; doi: 10.1093/molbev/msx241. <https://www.ncbi.nlm.nih.gov/pubmed/28961885>.

62. Simoes PM, Ingham RA, Gibson G, Russell IJ. A role for acoustic distortion in novel rapid frequency modulation behaviour in free-flying male mosquitoes. J Exp Biol. 2016;219 Pt 13:2039-47; doi: 10.1242/jeb.135293. <https://www.ncbi.nlm.nih.gov/pubmed/27122548>.

63. Poda BS, Cribellier A, Feugère L, Fatou M, Nignan C, Hien DFdS, et al. Spatial and temporal characteristics of laboratory-induced Anopheles coluzzii swarms: shape, structure and flight kinematics. bioRxiv. 2024:2024.03.25.586329; doi: 10.1101/2024.03.25.586329.

64. Fatou M, Müller P. 3D video tracking analysis reveals that mosquitoes pass more likely through holes in permethrin-treated than in untreated nets. Sci Rep. 2024;14 1; doi: <https://doi.org/10.1038/s41598-024-63968-y>.

65. Fatou M, Müller P. In the arm-in-cage test, topical repellents activate mosquitoes to disengage upon contact instead of repelling them at distance. Sci Rep, in press. 2024.

66. Kimura T, Ohashi M, Crailsheim K, Schmickl T, Okada R, Radspieler G, et al. Development of a new method to track multiple honey bees with complex behaviors on a flat laboratory arena. PLoS One. 2014;9 1:e84656; doi: 10.1371/journal.pone.0084656. <https://www.ncbi.nlm.nih.gov/pubmed/24465422>.
